# Investigating interactions between phentermine, dexfenfluramine, and 5-HT_2C_ agonists, on food intake in the rat

**DOI:** 10.1007/s00213-014-3829-2

**Published:** 2014-12-20

**Authors:** Andrew J. Grottick, Kevin Whelan, Erin K. Sanabria, Dominic P. Behan, Michael Morgan, Carleton Sage

**Affiliations:** Arena Pharmaceuticals Inc, 6154 Nancy Ridge Drive, San Diego, CA 92121 USA

**Keywords:** Synergy, BELVIQ®, Lorcaserin, Isobologram, Fen-phen

## Abstract

**Rationale:**

Synergistic or supra-additive interactions between the anorectics (dex)fenfluramine and phentermine have been reported previously in the rat and in the clinic. Studies with 5-HT_2C_ antagonists and 5-HT_2C_ knockouts have demonstrated dexfenfluramine hypophagia in the rodent to be mediated by actions at the 5-HT_2C_ receptor. Given the recent FDA approval of the selective 5-HT_2C_ agonist lorcaserin (BELVIQ®) for weight management, we investigated the interaction between phentermine and 5-HT_2C_ agonists on food intake.

**Objectives:**

This study aims to confirm dexfenfluramine-phentermine (dex-phen) synergy in a rat food intake assay, to extend these findings to other 5-HT_2C_ agonists, and to determine whether pharmacokinetic interactions could explain synergistic findings with particular drug combinations.

**Methods:**

Isobolographic analyses were performed in which phentermine was paired with either dexfenfluramine, the 5-HT_2C_ agonist AR630, or the 5-HT_2C_ agonist lorcaserin, and inhibition of food intake measured in the rat. Subsequent studies assessed these same phentermine-drug pair combinations spanning both the full effect range and a range of fixed ratio drug combinations. Satellite groups received single doses of each drug either alone or in combination with phentermine, and free brain concentrations were measured.

**Results:**

Dex-phen synergy was confirmed in the rat and extended to the 5-HT_2C_ agonist AR630. In contrast, although some synergistic interactions between lorcaserin and phentermine were observed, these combinations were largely additive. Synergistic interactions between phentermine and dexfenfluramine or AR630 were accompanied by combination-induced increases in brain levels of phentermine.

**Conclusions:**

Dex-phen synergy in the rat is caused by a pharmacokinetic interaction, resulting in increased central concentrations of phentermine.

## Introduction

Fenfluramine (Pondimin) and dexfenfluramine (Redux) are anorectic agents which act to enhance serotonergic transmission both through inhibition of 5-HT reuptake by the parent compounds, and through their major circulating des-ethylated metabolite, (dex)norfenfluramine, which is a 5-HT reuptake inhibitor, a 5-HT and noradrenaline releasing agent, and a potent agonist at postsynaptic 5-HT_2_ receptors (Curzon et al. 1997; Garattini et al. 1986; Mennini et al. 1991; Porter et al. 1999).

Both compounds were FDA-approved appetite suppressants until 1997, when they were withdrawn due to their association with cardiac valvular heart disease (Connolly et al. 1997; http://www.fda.gov/Drugs/DrugSafety/PostmarketDrugSafetyInformationforPatientsandProviders/ucm179871.htm). While in clinical use, it had become common practice for off-label co-administration of fenfluramines with the anorectic sympathomimetic phentermine (a combination commonly referred to as fen-phen). This co-administration was apparently driven by two distinct rationales (see Wellman and Maher 1999). Firstly, that low dose combinations of two drugs with distinct mechanisms of action that share a clinical effect may enable maintenance of efficacy, and thus the possibility of reduced, mechanism-based side effects. Secondly, it was thought that the stimulant phentermine may counteract sedation associated with fenfluramine use. Anecdotal data soon emerged from clinical experience that greater weight loss appeared to occur with the combination than would be expected with simply adding the efficacy of either agent alone (Weintraub et al. 1984). Clinical use of the combination subsequently exploded, such that between 1992 and 1997, it was estimated that nearly 18 million prescriptions were written for fen-phen (Kassirer and Angell 1998). The pharmacology of fen-phen on measures of food intake in the rat was subsequently tested (Roth and Rowland 1998, 1999; Wellman et al. 2003). These studies assessed the interaction in both deprived and fed rats eating standard lab chow or palatable sweetened milk after both acute and chronic dosing, essentially confirming suspicions from the clinic that the effect of the combination was more than the sum of its constituent parts.

At around the same time, studies using both selective and non-selective antagonists of the 5-HT_2C_ receptor demonstrated that at least in the rodent, the effects of dexfenfluramine on food intake were 5-HT_2C_ mediated (Neill and Cooper 1989; Grignaschi and Samanin 1992; Hartley et al. 1995; Curzon et al. 1997; Vickers et al. 2001). This was consistent with findings that a number of less than optimally selective 5-HT_2C_ receptor agonists such as mCPP and Ro 60–0175 reduced food intake and body weight in rodents (for review see Halford et al. 2005), that 5-HT_2C_ receptor null mice are hyperphagic and obese (Tecott et al. 1995), and that the hypophagic effects of dexfenfluramine are significantly attenuated in such animals (Vickers et al. 1999). Clinical studies also demonstrated that the 5-HT_2C_ receptor preferential agonist meta-chlorophenylpiperazine (mCPP) reduced hunger and food intake in healthy, normal-weight volunteers (Walsh et al. 1994) and hunger and body weight in the obese (Sargent et al. 1997). These data, coupled with the withdrawal of the fenfluramines in 1997, led to an industry-wide effort in the late 1990s to develop selective 5-HT_2C_ agonists which would be efficacious in the treatment of obesity and be devoid of the valvulopathy which was believed to be associated with 5-HT_2B_ activation (Fitzgerald et al. 2000; Rothman et al. 2000).

This effort led to the development of lorcaserin (BELVIQ®) which is now approved by the FDA for use in weight management in obese (BMI >30) or overweight (BMI 27–30) patients with a comorbid condition. Lorcaserin is a selective 5-HT_2C_ agonist devoid of activity at the 5-HT_2B_ receptor at therapeutically relevant concentrations (Thomsen et al. 2008; Unett et al. 2013), making it unlikely to affect heart valve function. This lack of impact was confirmed by an extensive development program of phase 2 and 3 clinical studies which included prospective echocardiographic assessments of valvular function (Weissman et al. 2013). Given that dexfenfluramine-induced weight loss is 5-HT_2C_ mediated, and that dex-phen effects appeared synergistic, the current studies were designed to assess whether other, more selective 5-HT_2C_ agonists may act in a similar, synergistic manner.

The aims of the present work were fourfold: firstly, to replicate previous findings in the literature suggesting synergistic interactions between dexfenfluramine and phentermine, and secondly to determine whether these findings extended to other agonists of the 5-HT_2C_ receptor. For this, we used two structurally distinct 5-HT_2C_ agonists: an internal standard from previous lead optimisation at Arena Pharmaceuticals, AR630, and the FDA approved medication lorcaserin (BELVIQ®). Various methods of measuring the efficacy of drug combinations have been described previously (see Tallarida 2001). We initially investigated the relationship of these combinations using a standard isobolographic method, in which doses of each individual compound producing a given effect (typically the ED_50_) are compared with combinations of the two drugs together which produce the same effect. In order to assess the robustness of these results, our third aim was to compare the standard isobologram with a more detailed response-surface analysis as described previously (Tallarida et al. 1999). This method simultaneously assesses drug interactions across effect levels and at different concentration ratios. Fourthly, we investigated whether any observed synergy could be explained by drug interactions at the pharmacokinetic level by measuring brain concentrations of all drugs in the presence and absence of phentermine.

As reported previously, we confirm that dexfenfluramine and phentermine act synergistically to reduce food intake in the rat and extend this observation to the 5-HT_2C_ agonist AR630. In contrast, although some synergistic interactions between lorcaserin and phentermine were observed, these combinations were largely additive. We further demonstrate that the synergy observed with dexfenfluramine and AR630 is most likely driven by increases in central phentermine exposure as a result of pharmacokinetic drug-drug interactions.

## Materials and methods

All animal procedures were performed according to protocols approved by the Arena Pharmaceuticals Animal Care and Use Committee following NIH guidelines.

### Drugs

**Fig. 1:**
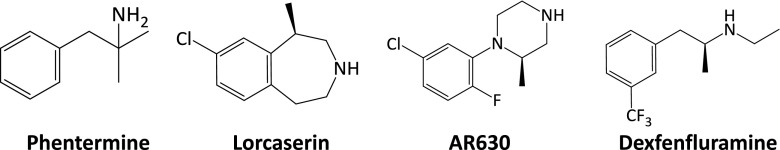
Chemical structures of compounds used in the studies

Lorcaserin HCl hemihydrate and AR630 were synthesized at Arena Pharmaceuticals Inc. AR630 is a 5-HT_2C_ agonist which is structurally distinct from lorcaserin (Fig. 1), although its 5-HT_2C_ agonist potency and selectivity is very similar^1^. Phentermine HCl and (S)+-fenfluramine (dexfenfluramine) were purchased from Sigma Aldrich. All compounds were dissolved in 20 % hydroxypropyl-beta-cyclodextrin (in sterile water, *w*/*v*) and were administered PO in a volume of 1 mL/kg. For combination treatments, lorcaserin, AR630, and dexfenfluramine powder were dissolved in phentermine solutions of appropriate concentrations. All doses are expressed as those of the free base.

### Food intake studies

#### Animals and housing

Male Sprague–Dawley rats (Harlan, San Diego, CA) weighing 250–350 g were used for behavioural studies. Upon arrival at the test facility, animals were triple housed within a holding room controlled for humidity, temperature, and light (lights off 1800–0600 hours). Rats received food (2018SX Teklad Rodent Diet) and water ad libitum unless stated otherwise. Over the course of all studies, rats were re-used up to four times, with at least one week of washout between tests. Rats were assigned to treatment groups pseudo-randomly from test to test. One week prior to initial drug testing, all rats were subject to a single food intake test which was identical to all subsequent drug tests, except that all rats received vehicle administration. Group size for all studies, including brain sampling, was eight.

#### Food intake measurement

Eighteen hours prior to food intake testing (1600 hours), food was removed from home cages. On the next day, animals were weighed and placed into cages with grid floors at 1000 hours, and allowed to acclimate to these cages for a 90-min period with free access to water and no food. At 1130 hours, rats were injected with compounds via oral gavage. Thirty minutes after injection (1200 hours), animals were allowed access to food. Food was then weighed at 30 min after food exposure (1 h after drug administration). Upon test conclusion, rats were returned to home cages with ad libitum access to food and water.

### Measurement of tissue drug concentrations

In vivo methods employed for pharmacokinetic studies were identical to those described for food intake studies except that after dosing, rats were not placed into new cages with grid floors. At 60 min after dosing (and 30 min after food access), rats were anesthetized with isoflurane, blood was collected via cardiac puncture, and brains were removed from the cranium. Blood samples were dispensed into sodium heparinized vials and capped and stored at 4 °C. Plasma was separated from formed elements in blood by centrifugation (10 min at 3,000×*g*) and frozen. Brains were rinsed with ice-cold phosphate-buffered saline, blotted dry, weighed, and frozen. Plasma and brain samples were stored at −80 °C prior to bioanalytical analysis.

#### Bioanalytical method

Brain samples were thawed on ice, placed into 50 mL plastic conical tubes, and two volumes of purified water/gram of brain was added. Brains were homogenized using a mechanical variable speed tissue homogenizer; 50 μL of brain homogenate was transferred to a 1-mL plastic tube. A volume of acetonitrile (200 μL) containing internal standard was added to brain homogenate, mixed by vortex, and centrifuged (15 min at 3,700 rpm). Standard curves (from 1 to 2,500 ng/mL) and quality control samples (6, 60, 600 ng/mL) were prepared in blank rat brain in a similar manner. Supernatants were analysed for lorcaserin, AR630, phentermine, dexfenfluramine, and the major metabolite of dexfenfluramine, norfenfluramine. Compound concentrations were determined using a LC-MS/MS method. Analytes were separated by reverse phase chromatography (Kinetex 3 × 30 mm, C18, 2.6 μm, 100 Å, HPLC Column, Phenomenex, Torrance, CA) using a binary gradient on a Shimadzu LC system (Shimadzu Corp., Columbia, MD). The gradient consisted of 0.1 % formic acid in water and 0.1 % formic acid in acetonitrile with a flow rate of 650 μL/min. Detection of lorcaserin, phentermine, dexfenfluramine, norfenfluramine, and internal standards was achieved with electrospray ionization (TurboIonSpray) in positive ion mode with an API 5000 LC/MS/MS detector, (Applied Biosystems, Foster City, CA) using MRM transitions of 196.1/144.2 Da (lorcaserin), 232.0/187.0 Da (dexfenfluramine), 150.0/133.0 Da (phentermine), 204.1/187.0 Da (Norfenfluramine), and 202.1/149.1 Da (lorcaserin-d6, internal standard).

#### Study 1, isobolograms

Dexfenfluramine (0.25–4 mg/kg), lorcaserin (0.5-8 mg/kg), phentermine (0.5-8 mg/kg), and AR630 (1–16 mg/kg) were assessed for inhibition of food intake. Doses were selected based upon previous in-house experience with these compounds and ascended in twofold steps. Two subsequent studies were run for each isobologram, with dose–responses of each compound pair run in combination with a fixed, low dose of the other (phentermine 1 mg/kg; AR630 1 mg/kg; dexfenfluramine 0.5 mg/kg; lorcaserin 1 mg/kg). To approximate the true experimental errors in these experiments, a bootstrapping with replacement approach was used (Huber 1981; Launer and Wilkinson 1979) in which five data sets were generated from random samples of the measured data points for both the individual dose–responses and for each drug/dose combination. These were used to generate ED_50_ values and 95 % confidence limits for all datasets which were subsequently plotted in an isobologram. A line of additivity with confidence limits was then drawn between the dose–responses of each compound alone.

#### Study 2, response-surface analysis

Methods are described in detail elsewhere (Tallarida et al. 1999). Briefly, percent inhibition relative to mean vehicle intake was calculated for each rat in drug-treated groups from the dose–response studies and logarithmic regressions applied. Resultant equations were used to calculate relative potencies (*R*) of compound pairs (lorcaserin-phentermine, dexfenfluramine-phentermine, and AR630-phentermine) across the effect range (10–90 % inhibition), with *R* values at the ED_50_ used as a basis for dose selection in subsequent studies: Three separate studies for each compound pair were then conducted, with ratios of doses selected based upon *R*/2, *R*, and 2*R*. Actual doses were based upon assumptions both from the isobolographic analysis and previous experience with these drug combinations. These were designed to capture the full dose–effect range (see Table 1, columns 2 and 3).

**Table 1:** Combination dose–effect data and calculated quantities for AR630, dexfenfluramine, and lorcaserin in combination with phentermine at three fixed ratio combinations. *R*_calc_, relative potencies of the two drugs at the percent inhibition achieved; *A*_eq_, equivalent dose of compound A represented by phentermine dose; *A*_corr_, dose of compound A expected from dose–response studies to produce percent inhibition achieved; *α*, interaction index where *α* < 1 = more than additive

	Dose combinations (mg/kg)						
	AR630	Phentermine	%	*R*_calc_	*A*_eq_	*A*_corr_	*α*
Set 1	0.27	0.32	3.44	1.39	0.72	1.07	0.71*
*R* = 0.85	0.48	0.56	23.25	1.46	1.30	1.52	0.72**
	0.85	1.00	60.91	1.70	2.55	4.75	0.47**
	1.51	1.78	86.54	1.92	4.93	12.56	0.41**
	2.69	3.16	98.82	2.01	9.05	18.09	0.50**
						$\overline{\mathbf{x}}$	**0.56**
Set 2	0.41	0.24	30.61	1.55	0.78	2.39	0.40**
*R* = 1.7	0.71	0.42	40.91	1.61	1.39	3.25	0.47**
	1.28	0.75	73.15	1.83	2.65	8.45	0.33**
	2.26	1.33	88.15	1.92	4.82	12.65	0.36**
	4.03	2.37	95.01	1.99	8.73	16.16	0.55**
						$\overline{\mathbf{x}}$	**0.42**
Set 3	0.58	0.30	13.78	1.45	1.01	1.45	0.72**
*R* = 3.4	1.02	0.53	37.79	1.59	1.86	2.96	0.72
	1.80	0.95	64.68	1.77	3.48	6.57	0.56**
	3.20	1.69	83.27	1.90	6.41	11.40	0.60**
	5.71	3.00	92.66	1.95	11.55	13.87	0.78**
						$\overline{\mathbf{x}}$	**0.67**

	Dose combinations (mg/kg)						
	Dexfenfluramine	Phentermine	%	*R*_calc_	*A*_eq_	*A*_corr_	*α*
Set 1	0.09	0.32	7.11	0.30	0.19	0.21	0.89
*R* = 0.3	0.17	0.56	19.41	0.36	0.37	0.41	1.07
	0.30	1.00	38.51	0.42	0.72	0.68	0.96
	0.53	1.78	66.55	0.64	1.68	2.44	0.71**
	0.95	3.16	98.96	0.93	3.89	7.89	0.48**
						$\overline{\mathbf{x}}$	**0.82**
Set 2	0.14	0.24	–	–	–	–	–
*R* = 0.6	0.25	0.42	19.08	0.36	0.40	0.41	1.12
	0.45	0.75	40.61	0.47	0.80	0.92	1.13
	0.80	1.33	69.57	0.67	1.69	2.74	0.69*
	1.42	2.37	97.16	0.92	3.61	7.73	0.47**
						$\overline{\mathbf{x}}$	**0.85**
Set 3	0.20	0.17	16.73	0.35	0.26	0.37	0.99
*R* = 1.2	0.36	0.30	30.38	0.41	0.48	0.63	0.90
	0.64	0.53	58.09	0.58	0.94	1.78	0.68
	1.13	0.94	71.57	0.64	1.73	2.38	0.63**
	2.01	1.68	98.84	0.93	3.57	7.94	0.44**
						$\overline{\mathbf{x}}$	**0.73**

	Dose combinations (mg/kg)						
	Lorcaserin	Phentermine	%	*R*_calc_	*A*_eq_	*A*_corr_	*α*
Set 1	0.13	0.32	5.04	0.76	0.37	0.61	0.63**
*R* = 0.42	0.24	0.56	0.16	0.75	0.66	0.53	1.31
	0.42	1.00	28.91	0.82	1.24	1.20	1.19
	0.75	1.78	58.03	0.88	2.32	2.73	0.94
	1.33	3.16	83.17	0.95	4.32	5.57	0.81
						$\overline{\mathbf{x}}$	**0.98**
Set 2	0.20	0.24	8.41	0.77	0.38	0.67	0.65*
*R* = 0.86	0.35	0.42	4.58	0.76	0.67	0.60	1.06
	0.63	0.75	29.89	0.82	1.24	1.23	1.11
	1.12	1.33	46.61	0.86	2.26	1.98	1.45
	1.99	2.37	68.45	0.91	4.15	3.67	1.17
						$\overline{\mathbf{x}}$	**1.09**
Set 3	0.50	0.30	15.20	0.78	0.74	0.81	0.87
*R* = 1.68	0.90	0.53	27.97	0.81	1.33	1.17	1.17
	1.59	0.95	49.88	0.86	2.42	2.17	1.14
	2.83	1.69	75.39	0.93	4.40	4.47	1.07
	5.04	3.00	96.69	0.98	7.99	8.17	0.95
						$\overline{\mathbf{x}}$	**1.04**

The interaction index (*α*) was then calculated for percent inhibition associated with each dose–pair combination: since *R* differed according to percent inhibition of food intake for all three compound pairs, logarithmic regressions were derived for *R* across the effect range. These equations (*R* ^2^ = 1 in all cases) were used to calculate the *R* associated with each percent inhibition for each subject, which in turn was used to calculate the equivalent dose level of either dexfenfluramine, lorcaserin, or AR630 (*A*_eq_) to produce that effect. The actual dose (*A*_corr_) of compound that produced that effect was then calculated from the initial dose–response functions and compared with *A*_eq_ to provide *α*, where *α* < 1 suggests synergy.

### Data analysis

To assess whether mean *α* values differed from 1 (no synergy) in study 2, *z* scores were generated where [*z* = (observed mean − expected mean (1))/standard error] and compared with the appropriate t statistic (*t*(8) = 2.31, 3.36 for *p* = 0.95 and 0.99, respectively; Snedecor and Cochran 1989). Whether alpha changed as a function of drug ratio was assessed with a one-way ANOVA with *R* as a factor, and the role of effect level in drug interactions assessed by plotting percent inhibition of food intake against alpha and performing a linear regression. Lastly, brain drug concentrations were compared in the presence or absence of the other analysed by one-way ANOVA with combination treatment as a factor.

## Results

### Study 1, isobolographic analysis

**Fig. 2:**
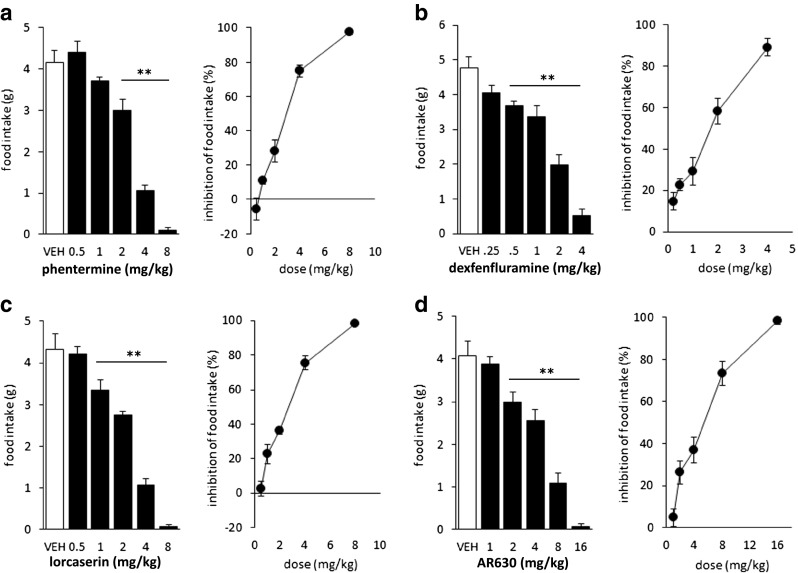
Effect of oral administration of phentermine (**a**), dexfenfluramine (**b**), lorcaserin (**c**), and AR630 (**d**) on food intake in the rat. *Left panels*, absolute intake after various doses; *right panels*, associated percent inhibition. **p < 0.01 versus vehicle groups

**Fig. 3:**
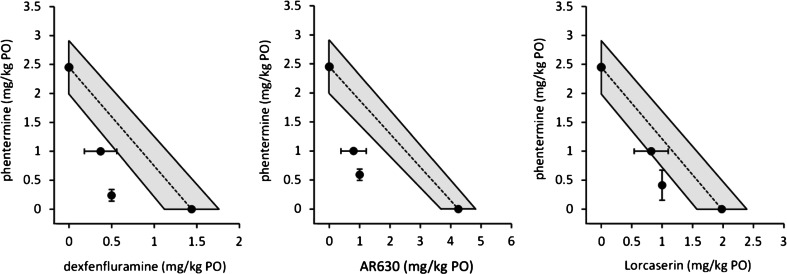
Isobolographic plots for the anorectic effects at ED_50_ of three phentermine-drug combinations. *Left panel*, dexfenfluramine; *middle panel*, AR630; *right panel*, lorcaserin. *Dotted lines* and *shaded areas* represent the predicted lines of additivity and associated 95 % confidence intervals for all drug pairs. Additional points represent ED_50_ values and associated 95 % confidence intervals derived from studies in which phentermine (1 mg/kg) was combined with various doses of the other compounds or where fixed doses of dexfenfluramine, AR630, or lorcaserin were combined with various doses of phentermine

Phentermine, AR630, dexfenfluramine, and lorcaserin all dose-dependently reduced food intake in the rat (Fig. 2), with selected dose ranges effectively covering the full range of effect. Calculated ED_50_ values derived from logarithmic regressions were 2.45, 4.25, 1.44, and 1.98 mg/kg for phentermine, AR630, dexfenfluramine, and lorcaserin, respectively. These values and their associated 95 % confidence intervals were plotted for each of the phentermine-drug combinations, and a line drawn between the two points to form isobolograms with a line of predicted additivity with associated confidence intervals (Fig. 3). Subsequent studies in which fixed low doses of each drug were combined with a dose–response of the other drug from each combination yielded ED_50_ values and associated 95 % confidence limits which were added to the isobolograms. Visual inspection of these indicated synergy for all the dex-phen and AR630-phen combinations, and for the two lorcaserin-phentermine combinations one additive and one synergistic interaction.

### Study 2: response-surface analysis

Mean *α* values for the dose pairs derived from all studies were 0.57, 0.83, and 1.04 (AR630-, dexfenfluramine-, lorcaserin-phentermine combinations, respectively, Table 1). *z* scores derived for each drug pair demonstrated all combinations of AR630 and phentermine to differ significantly from 1, nearly half of the dexfenfluramine-phentermine combinations, and two of the 15 lorcaserin-phentermine combinations. Both of the significant *α* values for the lorcaserin-phentermine combination were associated with percent inhibition less than 10 %. ANOVA revealed no effect of the ratio of drug combinations on *α* for either dexfenfluramine- or lorcaserin-phentermine combinations [*F*(2,12) = 0.5 for both, NS] and a trend for AR630-phentermine which failed to reach statistical significance [*F*(2.12) = 3.4, *p* = 0.07]. No relationship was found between inhibition of food intake and *α* for the lorcaserin-phentermine combination (*R* ^2^ < 0.01, *F*(1,14) = 0.05, NS), a trend for the AR630-phentermine combination (*R* ^2^ = 0.16, *F*(1,14) = 2.47, *p* = 0.13), and a strong correlation for dexfenfluramine-phentermine (*R* ^2^ = 0.85, *F*(1,14) = 72.3, *p* < 0.01).

### Drug concentrations

Administration of all compounds resulted in brain concentrations with low variability between animals, and within expected ranges (Table 2), although based on the single 60-min timepoint, rate of formation of norfenfluramine from dexfenfluramine appeared to differ somewhat from other published works (Mennini et al. 1985; Garattini et al. 1979). Neither AR630 [*F*(1.14) = 0.1, NS], lorcaserin [*F*(1,14) = 0.2, NS], nor dexfenfluramine [*F*(1,14) = 0.1, NS] brain concentrations differed when they were administered alone or as a mixture with phentermine. Similarly, measured concentrations of the major circulating metabolite of dexfenfluramine, (dex)norfenfluramine which were approximately sixfold that of the parent, did not differ when dexfenfluramine was administered alone or with phentermine [*F*(1,14) = 1.0, NS].

**Table 2:** Brain concentrations of AR630, dexfenfluramine, lorcaserin, and phentermine measured 60 min after a single oral dose either alone or in combination with phentermine. Right hand column represents the ratio of drug concentrations when administered in a combination versus being administered alone. In the dexfenfluramine study, the major circulating metabolite of dexfenfluramine, norfenfluramine, was also measured. All concentrations are mean ± SEM, nanogram per gram. **p < 0.01: concentration measured in the combination versus concentration measured after a single dose

Compound (mg/kg PO)	Brain concentration (ng/g)		Fold-change in concentration
	Alone	Combination	
Phentermine (1)	226 ± 32	866 ± 120**	3.80**
AR630 (1)	82 ± 14	92 ± 25	1.12
Phentermine (1)	298 ± 29	787 ± 57	2.60**
Dexfenfluramine (0.5)	130 ± 13	141 ± 19	1.08
Norfenfluramine (−)	**805** **±** **50**	**760 ± 27**	**0.94**
Phentermine (1)	298 ± 29	335 ± 18	1.12
Lorcaserin (1)	1,375 ± 82	1,444 ± 66	1.05

This contrasts with brain concentrations of phentermine when administered alone or in combination with other drugs: Combining AR630 with phentermine led to a 3.8-fold increase in brain concentrations of phentermine when compared to administration alone [*F*(1,14) = 26, *p* < 0.01], and combining dexfenfluramine with phentermine led to a 2.6-fold increase [*F*(1,14) = 59, *p* < 0.01]. These changes were not driven by differential access to the central compartment, as brain/plasma ratios of phentermine remained at approximately 13 when dosed alone or in the various combinations (data not shown). In contrast, lorcaserin did not significantly alter phentermine concentrations in the brain (1.1-fold difference: *F*(1,14) = 1.2, NS).

## Discussion

Synergistic interactions between fenfluramine and phentermine in the rat have been described previously (Roth and Rowland 1998, 1999; Wellman et al. 2003), although only one of these studies formally tested the interaction. In this study, Roth and Rowland (1999) performed isobolographic analyses of sweetened milk intake after fen-phen administration, finding synergy which was more robust in the fed than the fasted state. Here, we recapitulate these data in deprived rats eating standard lab chow, demonstrating statistically significant synergistic interactions. We also performed isobolographic analyses on phentermine combinations with two structurally distinct 5-HT_2C_ agonists, finding robust synergy with one (AR630), and in the two interaction studies with lorcaserin and phentermine both a moderate synergistic effect and simple additivity. In order to further probe these interactions, we pursued an additional method for assessing drug interactions (Tallarida et al. 1999). Using this response-surface analysis, we confirmed synergy with both dexfenfluramine and AR630 in combination with phentermine. Correlating percent inhibition of food intake with the degree of interaction (*α*) revealed inverse relationships for dexfenfluramine, such that as inhibition of food intake (and thus dose) increased, so did synergy: In the dex-phen combination, only the *α* values associated with the two highest dose pairs in each study differed significantly from 1. These interactions contrast with lorcaserin, which in these response-surface studies were largely additive: Two of 15 *α* values differed from one, and these were both associated with low effect levels where one would expect higher relative error. Given that the anorectic activity of dexfenfluramine (Neill and Cooper 1989; Grignaschi and Samanin 1992; Hartley et al. 1995; Curzon et al. 1997; Vickers et al. 2001) and the 5-HT_2C_ agonists (Thomsen et al. 2008) are 5-HT_2C_ mediated, the differential findings between the three compounds in combination with phentermine argue against any simplistic mechanistic interaction, either at the receptor level or downstream thereof. We therefore sought to assess potential pharmacokinetic interactions between the drug-phentermine combinations as explanations for the interactions observed. Using doses of the individual compounds employed in the isobolographic analyses, we found that co-administration of phentermine with either dexfenfluramine or AR630 led to significantly higher concentrations of phentermine (2.6- and 3.8-fold, respectively) in brain than when dosed alone, whereas in combination with lorcaserin, levels of phentermine were unchanged. In contrast, concentrations of dexfenfluramine, AR630, and lorcaserin did not differ whether dosed alone or in combination with phentermine. This demonstrated that where synergy did occur it was accompanied by significant increases in central levels of phentermine elicited by the drug combinations. The increased efficacy of dexfenfluramine and AR630 in combination with phentermine was therefore associated with pharmacokinetic interactions. The fact that synergy with dexfenfluramine was more apparent as dose increased is perhaps consistent with altered drug concentrations being responsible for the synergy. Additional studies would be needed to delineate the precise dose–effect relationship for pharmacokinetic interactions.

Other behavioural and neurochemical endpoints have been investigated with fen-phen in the rodent, including studies of central neurotransmitter release (Rada and Hoebel 2000; Balcioglu and Wurtman 1998; Shoaib et al. 1997), pain reactivity (Wellman 2008), models of cocaine addiction (Rothman et al. 1998; Glatz et al. 2002; Glowa et al. 1997), alcohol consumption (Halladay et al. 2000), and fenfluramine-induced neurotoxicities (Halladay et al. 1998). Some, but not all of these have demonstrated synergistic interactions, and this is perhaps not surprising given that for some of these endpoints, such as conditioned place preference (Rothman et al. 1998), dexfenfluramine and phentermine produce an opposite pattern of effect which may cancel each other out. For other endpoints, a PK explanation is clearly incompatible. For example, Wellman (2008) reported a dose–responsive effect of phentermine on pain reactivity in the hot plate assay, and mild effects of dexfenfluramine only at high doses, with isobolographic analyses demonstrating additive interactions between the two. Further research is clearly needed to understand these discrepancies.

The mechanism by which the pharmacokinetic interaction occurs is not immediately obvious. Phentermine has two major metabolites formed primarily by the action of the cytochrome P450 (CYP) enzyme 3A4. This enzyme itself is not extensively inhibited by phentermine, and 70–80 % of a dose is excreted as unchanged phentermine in urine when administered alone. Although dexfenfluramine and AR630 interact with various CYPs (von Moltke et al. 1998; Arena unpublished observations), no mechanisms of drug metabolism or clearance appear to differentiate dexfenfluramine and AR630 from lorcaserin or indicate a potential interaction with phentermine. The observed interactions were also not associated with altered penetration of the central compartment, as brain/plasma ratios remained consistent throughout all studies. It is worth noting that similar interactions of phentermine with other drugs have been observed in the clinic, as combining the antiepileptic medication topiramate with phentermine leads to a 42 % increase in phentermine area under the curve in humans (Qsymia Drug Label information).

Although widely prescribed, thorough clinical investigations into the safety and efficacy of fen-phen in comparison with its constituent medications are lacking, including an assessment of possible pharmacokinetic interactions. The only formal efficacy testing of fen-phen appears to have been performed by Weintraub and colleagues (Weintraub et al. 1984; Weintraub 1994). Only in the former study was a comparison made between the combination and monotherapies, and in this study different doses and daily treatment times of phentermine and fenfluramine were applied alone or in combination. The authors found that weight loss did not differ amongst treatment groups, although, interestingly, they report a ‘marked, sustained increase in total fullness (*in patients receiving the combination*) whereas mean results from participants receiving the other active treatments remained near baseline and similar to the placebo response’. This suggests that the combination was producing an effect distinct from that produced by either product alone, again arguing against a simple pharmacokinetic explanation.

While the isobolographic method relatively unambiguously identified synergy between phentermine and both dexfenfluramine and AR630, the result was somewhat ambiguous with the lorcaserin-phentermine combination, with one point demonstrating synergy and the other simple additivity. The degree of potential synergy observed in any system may depend not only on the drugs themselves but also on their dose ratios (Tallarida et al. 1999). Therefore, in order to better understand this relatively ambiguous result, we decided to investigate the combination using a response-surface analysis. In the response-surface analysis, all lorcaserin-phentermine combinations were additive excepting two interaction terms. These occurred where percent inhibition of food intake was particularly low (<10 %) and therefore associated with high relative error, and additionally were not associated with a pattern of interaction either with dose ratio or efficacy, as in both cases the next, higher dose combination resulted in lower percent inhibition and the interaction terms exceeded one. The isobolographic method assumes a simple linear relationship between the ED_50_ values of two compounds, and deviation from this ‘line of additivity’ indicates synergy or sub-additivity (Breitinger 2012). This line essentially represents a planar slice through the response surface of two drugs but does not allow the investigation of synergy as a function of dose ratios. If the response surface is complicated, an isobolographic analysis may not allow the dissection of more subtle features of the drug interactions or synergy. This includes potential differences in relative potency of the two compounds across the effect level, thus not allowing for non-linearity. A response surface analysis is a more exhaustive investigation of the dose ratio dependence of synergy and may allow more subtle features of synergy to be observed and hypotheses against them to be developed to probe the mechanisms of synergy. Given that response-surface analysis studies require minimal additional resource, we therefore suggest that these should be considered.

Given the experimental methods required to reliably detect synergy, thorough investigation of synergistic drug interactions in the clinic is extremely challenging. More importantly, whilst synergy is a theoretically interesting proposition and a mechanism for significantly increased efficacy, synergistic interactions are not required for effective weight loss therapies. Body weight maintenance is a highly regulated physiological process mediated by multiple redundant systems, and as such weight loss induces compensatory mechanisms which work to oppose this loss (e.g. Ochner et al. 2013). Assuming that additional weight loss achieved through drug combinations is feasible and does not significantly increase side effects, whether this increase in weight loss is achieved through an additive, synergistic, or even sub-additive interaction is of little consequence to patients so long as absolute weight loss is increased. Therefore, if a combination of lorcaserin and phentermine results in more weight loss in humans compared to either lorcaserin or phentermine alone then it will be a welcome addition to the arsenal of pharmacotherapies for weight loss, regardless of the nature of the interaction in humans.

To summarize, these data suggest that when central drug concentrations are taken into account, interactions between phentermine, dexfenfluramine, and other 5-HT_2C_ agonists are dose-additive when measuring inhibition of food intake in the rat. This analysis suggests that in order to investigate the nature of synergistic interactions properly, an understanding of the pharmacokinetic properties of compounds alone and in combination is essential.

## Footnotes

1 AR630 functional activity: EC_50_ at 5-HT_2C_ = 10 nM (100 % intrinsic activity (IA)); 5-HT_2A_ = 400 nM (60 % IA); 5-HT_2B_ = 1 μM (<10 % IA); binding affinity (^125^I-DOI binding): *K*_i_ at 5-HT_2C_ = 4 nM; 5-HT_2A_ = 120 nM; 5-HT_2B_ = 100 nM (internally generated data)
